# Regeneration Versus Granulation Tissue Healing in a Hopeless Mature Mandibular Molar Post-Endodontic Management: A 40-Month Follow-Up Case Report

**DOI:** 10.3390/dj14040243

**Published:** 2026-04-20

**Authors:** Elhassan Hassanein, Petra Gierthmuehlen, Almaha S. Algazlan, Dalia Kaisarly, Moataz Elgezawi

**Affiliations:** 1 Department of Conservative Dentistry, Periodontology and Endodontics, Universitätsklinikum Düsseldorf, 40215 Düsseldorf, Germany; elhassanhassanein.hassan@med.uni-duesseldorf.de (E.H.); petra.gierthmuehlen@med.uni-duesseldorf.de (P.G.); 2 Department of Clinical Dental Sciences, College of Dentistry, Princess Nourah Bint Abdulrahman University, Riyadh P.O. Box 84428, Saudi Arabia; 3 Department of Conservative Dentistry, Periodontology and Digital Dentistry, LMU University Hospital, Munich, Goethe Str. 70, 80336 Munich, Germany; dalia.kaisarly@med.uni-muenchen.de; 4 Department of Restorative Dental Sciences, College of Dentistry, Imam Abdulrahman Bin Faisal University, Dammam 31441, Saudi Arabia

**Keywords:** regeneration, granulation tissue healing, endo-perio lesions, Ledermix, periodontal healing, root canal treatment

## Abstract

**Objective:** To report a rare case of pulp space tissue growth in a mature mandibular molar with severe endo-periodontal involvement after conservative endodontic treatment and to discuss the possible biological explanations, including regeneration and granulation tissue healing. Severe endo-periodontal lesions are challenging, particularly as endodontic regeneration is usually observed in immature teeth, while revascularization in mature teeth, especially in cases of advanced periodontal disease, is rare, as demonstrated in this case. **Methods:** This study reports a rare case of tissue regeneration versus granulation tissue healing in the pulp space, occurring alongside periodontal healing, in a mature mandibular molar with necrotic pulp and severe periodontal involvement. A 52-year-old patient presented with a mature mandibular molar (tooth #19) exhibiting necrotic pulp with severe endo-periodontal involvement, including grade-3 mobility, tenderness to percussion, a 12 mm probing depth, and extensive periradicular radiolucency. The tooth was diagnosed with necrotic pulp and symptomatic apical periodontitis and was deemed hopeless, with extraction planned. **Results:** Following patient refusal, endodontic treatment was initiated, including cleaning, shaping, and placement of the intracanal medicament, Ledermix. The patient canceled the extraction due to symptom resolution and disappeared for 12 months. On return, the patient presented with spontaneous pain exacerbated by thermal stimuli, consistent with symptoms of irreversible pulpitis. Clinical examination revealed significant clinical and radiographic improvements, including reduced probing depth (3 mm), no mobility, resolution of apical translucency, radiographic findings suggestive of canal narrowing, and a positive pulp sensibility response. Re-entry elicited profuse bleeding with newly formed vital tissue beneath the medicament. Sodium hypochlorite irrigation failed to achieve hemostasis; inflamed tissue was removed; root canals were cleaned, shaped and obturated; and treatment was completed with placement of a permanent coronal resin composite restoration. A forty-month follow-up showed an asymptomatic tooth with clinical and radiographic healing. **Conclusions:** This case demonstrates that conservative endodontic management may result in favorable clinical and radiographic outcomes in mature teeth with severe endo-peroidontal involvement, influencing extraction decisions. It provides clinical evidence suggestive of tissue regeneration and periodontal healing in a mature tooth with necrotic pulp and severe periodontal compromise, challenging conventional prognosis. The observed pulp space tissue growth may be suggestive of regeneration; however, alternative explanations, including granulation tissue healing or repair processes, cannot be excluded. Healing by granulation tissue in the pulp space remains possible. Root canal treatment in advanced endo-perio lesions can yield favorable outcomes and may influence extraction decisions. Further clinical and histological studies are needed to clarify underlying mechanisms and optimize treatment strategies.

## 1. Introduction

Pulpal and periapical lesions occur when these tissues are inflamed or damaged, mostly due to dental caries progression or traumatic injuries. In mature teeth with necrotic pulp and apical periodontitis, root canal treatment (RCT) is the standard clinical management, supported by an excellent success rate. In immature teeth with incompletely formed root apices, apexification is a procedural option to allow complete root formation [1]. The aim is to eliminate infection in the root canal system and seal it against future reinfection. The drawback is sacrificing sound tooth structure, leading to an increased risk of fracture.

Endodontic-periodontal lesions present a particular clinical challenge due to the complex anatomical and biological interrelationship between the pulp and periodontal tissues, often complicating diagnosis, treatment planning and prognosis [2].

Regenerative endodontics is an alternative approach aiming at re-establishing pulp vitality, but is limited to immature teeth due to their wide apical foramina and high stem cell activity in young patients [1,3]. Favorable outcomes of regenerative endodontics involve eradication of apical periodontitis, continued root development and sometimes thickening of root canal walls [4]. Regenerative endodontic therapy involves two principal approaches: first, stem cell transplantation, where externally harvested stem cells are seeded onto scaffolding materials and introduced into the damaged pulp region [5,6,7]. The second approach, cell homing, relies on in situ recruitment of the body’s own stem cells. Biomaterial scaffolds infused with chemotactic signaling agents are directly delivered into the root canal. Both strategies aim to restore necrotic or irreversibly inflamed pulp tissue by encouraging pulp-like tissue formation, dentin deposition, revascularization, and reinnervation, ultimately reviving tooth vitality and function. Successful outcomes depend on adequate canal disinfection and an appropriately open apical foramen [7,8]. According to the American Association of Endodontists’ (AAE) coding for pulp regeneration, this procedure necessitates the intentional enlargement of the apical foramen and stimulation of intracanal bleeding to form a blood clot scaffold.

Endodontic regeneration is successful in immature young teeth [9]; however, in mature teeth with fully formed root apices and reduced regenerative potential, true regeneration remains exceedingly rare, and available evidence suggests that the tissue formed within the canal space often represents repair or granulation tissue rather than a functional dentin–pulp organ [10,11,12,13]. This is further complicated in periodontally compromised teeth, where the local environment might inhibit stem cell migration and healing [3,14,15,16,17,18]. However, regenerative endodontic procedures have been extended to include mature teeth with necrotic pulps and apical periodontitis. Promising outcomes of apical healing and subsidence of signs and symptoms have been reported in young individuals after regenerative endodontic procedures that included chemomechanical debridement of the canals, establishment of apical bleeding and placement of mineral trioxide aggregate (MTA) [13,19,20,21].

Despite increasing interest in regenerative endodontic approaches, there is limited evidence regarding spontaneous tissue growth within the pulp space of mature teeth with severe endo-periodontal involvement, particularly in cases where no intentional regenerative procedures are performed. The biological nature of such tissues remains unclear and may represent regeneration, repair or granulation tissue healing [1,13,22,23].

The current case uniquely demonstrates how a simple conservative endodontic procedure can lead to the resolution of signs and symptoms, healing of extensive periodontal involvement, and regeneration or repair through pulp space tissue formation in a mature molar, thus challenging the conventional view of such teeth as hopeless.

## 2. Case Report

### 2.1. Patient Information

This case is reported in line with CARE guidelines [15]; it does not require the approval of the research ethics committee. The patient had no active systemic diseases or medications, no allergy to drugs or local anesthesia and did not smoke.

### 2.2. Clinical and Radiographic Examination

A 52-year-old female patient presented with a chief complaint of tooth mobility and discomfort in the lower left posterior region. Clinical examination of tooth #19 with an extensive class-II resin composite restoration showed grade-3 mobility, grade-3 furcation involvement, tenderness to percussion, and an isolated 12 mm periodontal pocket with purulent discharge. Pulp vitality testing was negative with cold (Endo-Frost cold spray) and electric sensibility (Digitest) testing.

**Figure 1:**
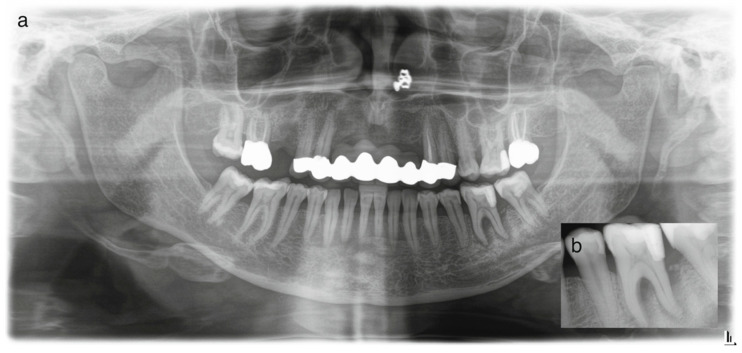
Panoramic radiograph at the initial visit (**a**); periapical radiograph at the initial visit displaying extensive periradicular radiolucency with evidence of bifurcation involvement (**b**).

Periapical and panoramic radiographic findings showed the following (Figure 1): tooth #19 showed advanced bone loss, periapical and periradicular radiolucency, an extensive class-II occlusodistal restoration, and intact root length with visible canal space. Teeth #3, 11 and 15 were endodontically treated, with two metal–ceramic crowns on teeth #3 and 15, and a fixed dental prosthesis including teeth #5, 6, 7, 8, 9, 10, 11 and 12, replacing missing teeth #7, 8, 9, and 10, as well as missing tooth #4 and resin composite restorations in teeth #14, 18, 19 and 31.

Clinical examination revealed a normal occlusal relationship with well-aligned arches and absence of both parafunctional habits and traumatic occlusion. The patient had generalized stage II grade-B periodontitis with localized stage III in teeth #12, 23, and 26, with pocket depths between 4 and 6 mm and CAL measuring ≥5 mm. The patient was under regular periodontal care by a periodontist. Oral soft tissues were free from lesions or abnormalities, and no mucogingival defects were found except for tooth #19. Extraoral examination was within normal limits.

According to AAE guidelines, the tooth was diagnosed as having a necrotic pulp with symptomatic apical periodontitis. Because of extensive periodontal involvement and mobility, the tooth was recommended for extraction and deemed hopeless. The patient refused extraction and insisted on undergoing conservative management to postpone extraction.

### 2.3. Procedure

A conservative endodontic protocol was applied to alleviate symptoms until extraction. Isolation was achieved using an Optradam Plus rubber dam system, which allowed clamp-free rubber dam isolation in this case, minimizing mechanical stress on the already mobile molar [24]. An access cavity was prepared under magnification using a microscope (Zeiss PROergo, Carl Zeiss AG, Oberkochen, Germany); four root canals were visible, and the working length was determined with an apex locator (Morita Root ZX mini, Morita, Fushimi-ku, Kyoto 612-8533, Japan) and confirmed with periapical radiographs. Canal lengths measured as follows: MB 21 mm, ML 20 mm, DB 22 mm and DL 21.5 mm. Canals were negotiated and shaped using Reciproc R25 (VDW, Munich, Germany). A standard irrigation protocol was followed using 17% EDTA and 3% sodium hypochlorite, both activated with an Eddy sonic device (VDW, Munich, Germany) for 1 min per canal, followed by final irrigation with 15 mL sodium hypochlorite and drying with paper points [25,26]. Ledermix paste (demeclocycline + triamcinolone) (Riemser, Greifswald, Germany) was placed in all canals, covered with a cotton pellet and glass ionomer (Ketac-Fil, 3M, Deutschland GmbH, Neuss, Germany) as a coronal restoration.

**Figure 2:**
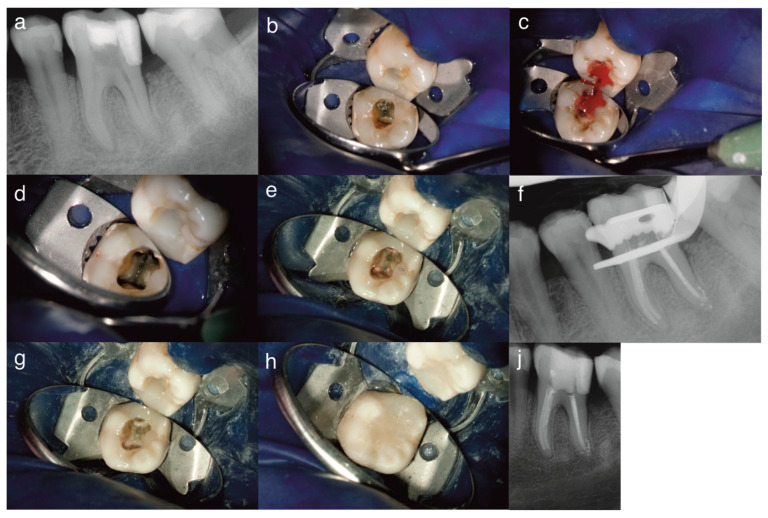
Periapical radiographs and images taken at the second visit after 12 months: (**a**) periapical radiograph with signs of periradicular healing and narrow root canals, (**b**) evidence of the intracanal medicament Ledermix after one year of placement, (**c**) vital tissue under the medicament with profuse bleeding indicating inflammation, (**d**) tooth after cleaning and shaping, (**e**) after root canal obturation, (**f**) periapical radiograph of root canal filling before coronal seal, (**g**) seal of the root canal orifices, (**h**) coronal resin composite restoration, and (**j**) post-operative periapical radiograph displaying periapical and periradicular healing.

After 12 months, the patient returned complaining of pulsating, spontaneous, dull pain exacerbated by cold or heat, pointing to the same tooth, and managing the pain with painkillers; the symptoms resembled irreversible pulpitis. The patient mentioned that after the first visit, symptoms subsided and she decided to cancel the extraction. The tooth showed an exaggerated response to cold lasting more than 30 s after stimulus removal, confirmed with electric pulp testing. Cold and electric pulp testing were performed in comparison to adjacent and contralateral teeth. There was no sensitivity to palpation or percussion, and no swelling or fistulous tracts. Periodontal examination revealed shallow periodontal probing depths (<3 mm), absence of tooth mobility and furcation involvement. Radiographs showed periradicular and periapical healing, narrowing of the root canal space, suggesting unplanned tissue regeneration in the pulp space (Figure 2).

According to AAE, at this stage, the tooth was diagnosed with normal periapical tissues. Upon re-entry under local anesthesia, vital tissues were observed within the canals under the intracanal medicament. Application of a pellet soaked in 3% sodium hypochlorite elicited profuse bleeding over 5 min (Figure 2). Due to signs and symptoms of irreversible pulpitis, the newly formed inflamed tissue was removed with hand K-files ISO 10 and 15 after confirming the working length with an apex locator. Reciproc files were then used for cleaning and shaping. Each canal was irrigated with 15 mL of 3% sodium hypochlorite, flushed with saline and dried with paper points [21].

RCT was decided, similar to Ahmed et al., where signs and symptoms resembling irreversible pulpitis had the decisive influence in performing RCT [21]. Obturation was performed with the lateral condensation technique using Reciproc 40 master points (VDW, Munich, Germany) and AH+ sealer (Dentsply, Sirona, Bensheim, Germany); the root canal filling was controlled by periapical radiograph (Figure 2).

The access cavity was cleaned with alcohol, and a self-etching adhesive (Clear-fill SE Bond, Kuraray, Tokyo, Japan) was applied and light-cured (40 s). Canal orifices were sealed with a flowable resin composite (Venus Flow, Kulzer, Wehrheim, Deutschland), light-cured for 40 s, covered with a nanohybrid composite (Filtek Supreme, Solventum, Seefeld, Germany) and light-cured for 60 s. A post-operative radiograph was taken (Figure 2).

### 2.4. Follow-Up

**Figure 3:**
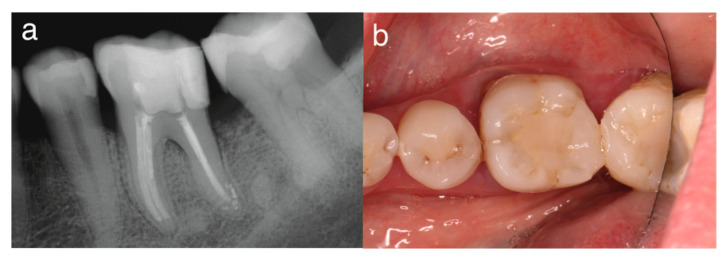
(**a**) Periapical radiograph and (**b**) occlusal view at 36-month follow-up.

**Figure 4:**
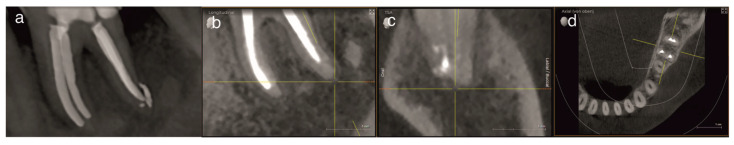
CBCT screenshots at different views and sections at the 40-month follow-up visit, showing (**a**,**b**) root canal filling short of the apices, and (**c**,**d**) apical tissue healing.

In the third visit, 36 months after the first visit, the patient showed no signs or symptoms and had normal periapical tissues (Figure 3). After 40 months, clinical examination confirmed healing of the periodontal tissues, with probing depths < 3 mm, no pain on palpation or percussion, and no fistulous tract or swelling. CBCT radiography confirmed apical tissue healing and the absence of missed extra canals (Figure 4).

## 3. Discussion

The relationship between the dental pulp and periodontium is intricate and changeable [27]. Endo-periodontal lesions complicate the practice, as they might necessitate interdisciplinary management and can affect the quality of treatment outcomes. The impact of pulpal disease on the periodontium is well-established, while the effect of periodontal lesions on the pulp is debatable [2,27]. In our case, communication between pulp and periodontium through furcation canals, accessory canals, or exposed dentinal tubules could have triggered the evident periodontal involvement in the initial presentation.

Although clinical regenerative endodontics targets immature teeth with incompletely formed root apices and devitalized pulps, there is an increased interest in regenerative endodontics in mature teeth with necrotic pulps as an alternative to routine RCT. Two cases were reported as successful, marked by resolution of periapical radiolucency and improvement in clinical signs and symptoms. The teeth had necrotic pulps and were mechanically instrumented to create large apical openings. The applied intracanal medication consisted of either calcium hydroxide or ciprofloxacin. After inducing bleeding into the canals by extending hand files beyond the apex, collagen membranes and MTA were placed [28]. Meanwhile, spontaneous periodontal healing, including periradicular and furcational bone, was reported in a case of advanced primary endodontic and secondary periodontic lesion after performing RCT on a mature mandibular molar [27].

Regenerative endodontics in mature teeth was investigated in young adults [21]. To the best of our knowledge, our report represents a rare case: the first documented instance of unexpected healing following conservative endodontic management of an extensive endo-periodontal involvement, with unexpected and unplanned tissue regeneration in the pulp space of a mature molar in a middle-aged patient. Notably, periodontal healing occurred in conjunction with tissue growth in the pulp space after initial chemomechanical debridement and placement of Ledermix medicament. This unusual combination of findings adds to the clinical significance of the case and supports its relevance for reporting unexpected periodontal healing. Because of extensive periodontal involvement and mobility, the tooth in question was considered hopeless. In cases planned for endodontic regeneration, intentional violation of the apical constriction to induce apical bleeding and apical clotting is a prerequisite procedure, as per AAE, which was not followed in our case. Initial endodontic intervention, involving cleaning and shaping followed by Ledermix application, apparently promoted regeneration of pulp space tissues or enhanced healing by granulation tissue and facilitated periodontal healing.

Since the initial intervention was done to alleviate pain until the time of extraction, no periodontal therapy was scheduled for the molar in question. The patient reported that, after improvement of symptoms following the first visit, she cancelled the extraction appointment, traveled abroad on a long trip and returned after 12 months with symptoms of irreversible pulpitis. The unexpected periodontal healing without any adjunctive periodontal therapy, observed after 12 months, might be due to minimizing the infection, reducing the transport of infectious agents, and promoting periodontal healing after the initial endodontic intervention during the first visit [1].

A clinical trial evaluated healing and pain following regenerative endodontics using platelet-rich plasma versus conventional RCT, with similar results in necrotic mandibular molars with apical periodontitis; thus, regenerative endodontics might be an alternative to RCT [21]. When signs and symptoms of irreversible pulpitis arose at any study intervals, an RCT was performed. A case report including a one-year follow-up in mature maxillary central incisors with necrotic pulp and symptomatic apical periodontitis presented an interesting outcome of regenerated tissue in the pulp space and resolution of apical periodontitis. Those teeth were treated with regenerative endodontics by inducing bleeding into the canal and MTA placement [13]. The regenerated pulp space tissue was histologically studied since the teeth that underwent traumatic injuries received RCT after 12 months. The regenerated vital tissues consisted of fibrous connective tissue that contained blood vessels, bone-like tissues and inflammatory cells.

In our case, we cannot assume that true pulp tissue regeneration occurred after the initial management, but it might represent either regenerated pulp space tissue or healing by granulation tissue [29]. Regaining sensitivity, as indicated by positive thermal and electric pulp testing during the second visit, was considered a functional criterion of vital pulp tissue and was previously reported to take place in very few cases of planned endodontic regeneration. However, no tissue sample from the pulp space was collected for histopathological examination. Although this constitutes a limitation of our case report, the significance of reporting this case should not be underestimated [29,30,31]. A histopathological examination of pulp space tissues would have provided insights into tissue response and healing mechanisms. Apical periodontitis may have elicited an apical resorption and widening of the apical foramina [32]. The tetracycline/corticosteroid mix in Ledermix, enhanced by previous irrigation with EDTA and sonic activation, may have acted synergistically to induce regeneration of pulpal tissues [33,34].

In the present case, the root canal obturation appeared slightly short of the radiographic apex. However, the working length was initially determined during the first visit using an electronic apex locator and confirmed with a radiograph. This finding may be attributed to changes in apical morphology during the healing process, including possible hard tissue deposition and narrowing of the root canal space, as observed radiographically at 12 months. Such anatomical alterations have been described in cases exhibiting intracanal tissue growth or repair processes, where dynamic changes in the apical region may occur over time. Therefore, the slightly short appearance of the obturation should be interpreted in the context of these biological changes rather than as a technical limitation [22,23].

Whether it is regeneration of pulp space tissues or only healing by granulation tissue, our case is the first documented instance of unintentional conservative endodontic management of a mature tooth with necrotic pulp and severe periradicular involvement. Ledermix is an endodontic medicament that has a dual anti-inflammatory effect because it contains corticosteroid, triamcinolone acetonide and an antibacterial effect as it contains demeclocycline hydrochloride (tetracycline-class broad-spectrum antibiotic). Ledermix, when used as an intracanal medicament, can promote healing and revascularization of necrotic pulps [34,35,36]. Moreover, irrigation with EDTA enhances the viability of dental pulp stem cells, promotes differentiation of odontoblasts and osteoblasts, facilitates their attachment to root canal walls, and stimulates pulp angiogenesis [37]. Sonic activation may have enhanced the effectiveness of EDTA irrigation in this context [25,26].

In the follow-up visit at 12 months, the patient reported dull aching pain and symptoms of irreversible pulpitis, which indicated unexpected tissue regenerative signs. The tooth had no mobility, and the probing depths had decreased significantly. Radiographically, the periradicular radiolucency had resolved, and there was evidence of canal narrowing suggestive of calcific deposition. The patient reported sensitivity and symptoms of irreversible pulpitis. Upon re-access, profuse bleeding indicated the presence of vital tissue, probable revascularization and regeneration of pulp space tissue, despite the original hostile periodontal conditions. RCT was decided because of the signs and symptoms resembling irreversible pulpitis. The management was similar to that of Ahmed et al., in their clinical trial of regenerative endodontics, where RCT was performed in cases of pain and disturbed healing at different study intervals [21]. Healing in their clinical trial was estimated clinically and radiographically, without performing any histopathological examination of the pulp space tissues in cases that developed signs and symptoms of disturbed healing and received RCT.

Spontaneous healing might have been supported by residual apical vasculature. Mere apical resorption due to apical periodontitis might have increased the apical foramen dimension, providing greater potential for angiogenesis of the pulp. Stem cells from the apical papilla can be a source of revascularization and regeneration of pulplike tissues in immature teeth, leading to the previously reported maturation, regeneration of pulplike tissues and thickening of dentin walls. However, the literature indicates that in mature teeth with complete apical closure, as in our case, the apical papilla is usually absent and therefore cannot contribute to the regeneration of tissues in the pulp space. Instead, stem cells from the periodontal ligament and bone marrow can contribute to tissue regeneration in the pulp space in the form of fibrous tissue and blood vessels, or bone-like or cementum-like tissues. Stem cells of the periodontium could have also participated in periodontal healing [38,39]. A study showed promising potential of using exosomes of apical papilla stem cells to induce the regeneration of pulplike tissues [40]. Since periodontal healing in the tooth in question was complete, there was no need to perform periodontal therapy beyond resuming the regular periodontal care that our patient used to follow.

The 36-month and 40-month follow-ups showed apical healing and no clinical signs and symptoms of apical periodontitis, indicating a successful RCT. The present case uniquely demonstrated that regenerative endodontic applications can be successful not only in immature teeth with incompletely formed root apices [41] but also in mature teeth with fully formed apices, even when periodontal involvement is extensive. Our case showed that regeneration of pulp space tissues and apical–periodontal healing might occur in older individuals [1,42,43].

## Data Availability

The original contributions presented in this study are included in the article. Further inquiries can be directed to the corresponding authors.
